# Maternal Zinc, Copper, and Selenium Intakes during Pregnancy and Congenital Heart Defects

**DOI:** 10.3390/nu14051055

**Published:** 2022-03-02

**Authors:** Jiaomei Yang, Yijun Kang, Qianqian Chang, Binyan Zhang, Xin Liu, Lingxia Zeng, Hong Yan, Shaonong Dang

**Affiliations:** 1 Department of Epidemiology and Health Statistics, School of Public Health, Xi’an Jiaotong University Health Science Center, Xi’an 710061, China; violetyjm18@xjtu.edu.cn (J.Y.); tjkyj@xjtu.edu.cn (Y.K.); cqq20160820@stu.xjtu.edu.cn (Q.C.); zhangbinyan@stu.xjtu.edu.cn (B.Z.); xinliu@xjtu.edu.cn (X.L.); tjzlx@xjtu.edu.cn (L.Z.); yanghonge@xjtu.edu.cn (H.Y.); 2 Key Laboratory of Environment and Genes Related to Diseases, Xi’an Jiaotong University, Ministry of Education, Xi’an 710061, China; 3 Nutrition and Food Safety Engineering Research Center of Shaanxi Province, Xi’an 710061, China

**Keywords:** zinc, copper, selenium, congenital heart defects, pregnancy

## Abstract

The effects of zinc, copper, and selenium on human congenital heart defects (CHDs) remain unclear. This study aimed to investigate the associations of the maternal total, dietary, and supplemental intakes of zinc, copper, and selenium during pregnancy with CHDs. A hospital-based case-control study was performed, including 474 cases and 948 controls in Northwest China. Eligible participants waiting for delivery were interviewed to report their diets and characteristics in pregnancy. Mixed logistic regression was adopted to examine associations and interactions between maternal intakes and CHDs. Higher total intakes of zinc, selenium, zinc to copper ratio, and selenium to copper ratio during pregnancy were associated with lower risks of total CHDs and the subtypes, and the tests for trend were significant (all *p* < 0.05). The significantly inverse associations with CHDs were also observed for dietary intakes of zinc, selenium, zinc to copper ratio, selenium to copper ratio, and zinc and selenium supplements use during pregnancy and in the first trimester. Moreover, high zinc and high selenium, even with low or high copper, showed a significantly reduced risk of total CHDs. Efforts to promote zinc and selenium intakes during pregnancy need to be strengthened to reduce the incidence of CHDs in the Chinese population.

## 1. Introduction

Congenital heart defects (CHDs) are the most common birth defects in the world, with an estimated birth prevalence of 9.4‰ [1]. CHDs remain the leading cause of morbidity, mortality, and disability in infancy and childhood [2] and can cause lifelong physical and mental comorbidities, imposing huge burdens on the family and society [2]. The estimated CHDs prevalence among live births is 9.0‰ in China, with more than 150,000 incident cases each year [3]. However, the potential mechanisms for most CHDs remain to be unclear. Therefore, it is important to identify modifiable risk factors to provide evidence for the primary prevention of CHDs.

Maternal nutrition during pregnancy, as an important modifiable factor, is critical for fetal development [4]. Animal studies have shown that prenatal zinc deficiency during gestation led to heart malformations in offspring [5]. Copper deficiency induced heart anomalies in rats [6], and copper excess induced high mortality and morphological malformations in the embryos and larvae of Pagrus major [7]. Maternal selenium deficiency was reported to relate to miscarriages, premature births, and intrauterine growth retardation [8,9,10]. However, there is little evidence from human studies about the effects of zinc, copper, and selenium on CHDs [11,12,13,14,15,16], and the results were not consistent [11,12,13,14,15,16]. One study reported an inverse association between zinc level and ventricular septal defects (VSD) [16], while another study found no associations of zinc level with CHDs and the subtypes [13]. There was even one study reporting higher zinc levels in the CHDs cases than in the healthy controls [11]. Two studies showed a positive association between copper level and CHDs [13,15], while the other two studies reported no association [14,16]. One study found an inverse association between selenium level and CHDs [12], while another study showed a positive association [14]. Thus, it is warranted to further conduct studies among humans to elucidate the effects of zinc, copper, and selenium on CHDs.

Diet and dietary supplements are the main sources of body zinc, copper, and selenium among pregnant women. As there are no reliable biological markers for zinc, copper, and selenium status, estimated usual intakes from the diet and supplements may be the optimal indicators of maternal zinc, copper, and selenium status for epidemiologic studies [17]. However, to our knowledge, there has been no available human study specifically exploring the associations between maternal intakes of zinc, copper, and selenium during pregnancy and CHDs. Moreover, complex antagonistic interactions among zinc, copper, and selenium have been reported in previous studies [18,19,20]. However, to our knowledge, there is only one published study involving the interactions of zinc and copper levels on CHDs [13]. Therefore, the current study aimed to investigate the relationships between maternal total, dietary, and supplemental intakes of zinc, copper, and selenium during pregnancy and CHDs among humans in Northwest China. The interactions among maternal intakes of zinc, copper, and selenium during pregnancy on CHDs were also explored in this study.

## 2. Materials and Methods

### 2.1. Study Design and Participants

Between August 2014 and August 2016, we performed a case-control study in six tertiary comprehensive hospitals in Xi’an City, Northwest China. The study design was previously published in detail [21,22]. Briefly, participants were enrolled among the pregnant women who were waiting for delivery in the obstetrics departments. These participants were all inhabitants of Northwest China and resided in Shaanxi, China, during pregnancy, where the trace element concentrations are not considerably different [23]. Mothers whose fetuses were diagnosed with isolated CHDs and had no chromosomal abnormalities or gene disorders were included in the cases, and mothers whose fetuses were diagnosed with no congenital malformations were included in the controls. Mothers with gestational diabetes or multiple gestations were excluded from this study because of potentially different etiologies. Because of the low birth incidence of CHDs, any eligible case mothers were included in the study without sampling methods. Specialists from the ultrasound, pediatrics, and obstetrics departments conducted the diagnoses of the cases and controls. The diagnostic criteria were standard and strictly enforced by these qualified specialists in each hospital. A telephone follow-up was also undertaken within one year after birth to confirm the diagnoses. All the CHDs diagnoses were ascertained by echocardiography and/or cardiac catheterization and/or surgery. The controls were randomly selected each month in each hospital, and the ratio of the number of cases to controls included in the same month in the same hospital was 1:2. On the assumptions of the estimated percentages of pregnant women taking foods rich in nutrients more than three times a week in cases and controls being 37.2% and 45.0%, respectively, the correlation of exposure between cases and controls being zero, the type I error rate 0.05, and the power of the test 80%, the sample sizes of cases and controls were 474 and 948, respectively.

This study was approved by the Xi’an Jiaotong University Health Science Center on March 2012 (No.2012008). Participants provided written informed consents. 

### 2.2. Dietary Assessment

Eligible women waiting for delivery in the hospital were interviewed to recall diets in the entire pregnancy through a 111-item semi-quantitative food frequency questionnaire (FFQ). The median time between interview completion and date of delivery was two days for both the cases and controls. Maternal dietary patterns and nutrient intakes tend to be stable across pregnancy [24,25]; thus, maternal dietary intakes during the whole pregnancy are comparable with those during the critical period of cardiac development in the 3rd-8th week of gestation [21,22,26]. The FFQ was established according to a validated FFQ used for pregnant women in Northwest China [27]. Pearson’s correlation coefficients for zinc, copper, and selenium between the FFQ and the average of six 24 h recalls were 0.67, 0.54, and 0.59, respectively, and for other nutrients, they ranged from 0.53 to 0.70 [27]. Women recalled consumption frequency according to eight predefined categories and reported portion sizes with the assistance of food portion images [28]. Participants also reported the type/brand and the number of dietary supplements taken and the number of days taking dietary supplements in each trimester of pregnancy. Daily nutrient intakes were derived by the Chinese Food Composition Tables [29,30]. The total intake of one nutrient was calculated as the sum of dietary and supplemental intakes.

### 2.3. Covariates

General information of the participants was collected face-to-face by a standard questionnaire. The study covariates included (1) socio-demographic characteristics: maternal age (≥30 years/<30 years), residence (urban/rural), maternal education (senior high school or above/junior high school or below), maternal work (farmers/others), and parity (≥1/0); (2) maternal health-related factors in the first trimester: folate/iron supplements use (no/yes), passive smoking (no/yes), medication use (no/yes), and anemia (no/yes); and (3) dietary diversity score: the sum of ten food groups scores according to the FAO Minimum Dietary Diversity for Women guideline [31], with each group assigned a score of 1 if consumed and 0 if not consumed [26]. The ten food groups consist of starchy staple foods, pulse, nuts and seeds, dairy products, flesh foods, eggs, dark green leafy vegetables, vitamin A-rich fruits and vegetables, other vegetables, and other fruits [31]. Women having no paid employment outside their homes were classified as farmers. Passive smoking was defined as exposure to another person’s tobacco smoke for more than 15 min/d. Anemia during the first trimester was diagnosed by the physicians using the criteria of hemoglobin concentration lower than 110 g/L.

### 2.4. Statistical Analysis

In univariate comparisons, categorical variables were compared between groups using the χ^2^ test, and continuous variables were compared between groups using Mann–Whitney *U* test because of the non-normal distributions observed according to the Shapiro–Wilk test. Considering the clustering in the design through hospitals, we used mixed logistic regression models to estimate ORs (95%CIs) for total CHDs and CHDs subtypes associated with total and dietary intakes of zinc, copper, selenium, zinc to copper ratios, selenium to copper ratios, and zinc to selenium ratios and maternal zinc, copper, and selenium supplements use during pregnancy. Total and dietary intakes were divided into four categories according to the quartiles of the control distribution. Total and dietary zinc, copper, and selenium intakes were also categorized by the recommended nutrient intakes (RNIs) for Chinese pregnant women, which were 9.5 mg/d, 0.9 mg/d, and 65 mg/d, respectively [32]. Maternal zinc, copper, and selenium supplements uses were considered as binary categories (yes/no) because of the low amount of intake. The socio-demographic characteristics (maternal age, residence, education, work, and parity), maternal health-related factors in the first trimester (folate/iron supplements use, passive smoking, medication use, and anemia), and dietary diversity score were chosen as confounders in the models because they were reported to be associated with CHDs [26,33,34] and changed the estimates by more than 10% [35]. Since the intake of each mineral was highly correlated to the others, we did not mutually adjust for the intake of each mineral to avoid multicollinearity in the models [36]. Maternal supplements use and dietary intake of specific minerals were additionally mutually adjusted. *P* for trend was calculated by including quartile specific median intake in the model. To further explore the shape of the significant associations of total and dietary intakes with total CHDs, we used restricted cubic splines with three knots in the fully adjusted models. Moreover, we evaluated the interactions by introducing cross-product terms into regression models to assess whether the associations were modified by maternal age, residence, education, work, and folate/iron supplements use during the first trimester.

We evaluated the interactions between zinc and copper, selenium and copper, and zinc and selenium by introducing cross-product terms into regression models. We also re-categorized total zinc, copper, and selenium intakes as “low” and “high” according to the medians in the controls and evaluated the risk of total CHDs associated with high or low intakes of total zinc, copper, and selenium by mixed logistic regression using the combination of low intake as the reference.

The statistical analyses were performed using the Stata software (version 15.0; StataCorp, College Station, TX, USA). All tests were two-tailed with *p* < 0.05 considered statistically significant.

## 3. Results

### 3.1. Basic Characteristics of the Study Sample

Case mothers were less likely to reside in urban areas, have higher educational levels, work outside, and be nulliparity compared to the controls (Table 1). Folate/iron supplements use in the first trimester was more common in the controls than in the cases, while passive smoking, medication use, and anemia in the first trimester were more common in the cases than in the controls. Case mothers had lower dietary diversity scores and total energy intake in pregnancy than the controls. Moreover, case mothers had significantly lower total and dietary intakes of zinc, copper, selenium, zinc to copper ratios, and selenium to copper ratios during pregnancy than the controls. There were no differences in maternal age, neonatal gender, and total and dietary zinc to selenium ratios between the two groups. The percentages of case mothers having total zinc, copper, and selenium intakes below the RNIs for Chinese pregnant women were 85.7%, 15.2%, and 97.1%, respectively, which were all higher than those in control mothers (67.4%, 8.4%, and 88.3%, respectively).

### 3.2. Maternal Total and Dietary Zinc, Copper, and Selenium Intakes during Pregnancy and CHDs

When comparing the quartile 4 (highest), quartile 3, and quartile 2 to the quartile 1 (lowest) of total zinc intake, the fully adjusted ORs (95%CIs) for total CHDs were 0.22 (0.12–0.42), 0.57 (0.36–0.91), and 0.65 (0.45–0.94), respectively, and the test for trend was significant (*p* < 0.001) (Table 2). When comparing the quartile 4, quartile 3, and quartile 2 to the quartile 1 of total selenium intake, the fully adjusted ORs (95%CIs) for total CHDs were 0.29 (0.15–0.54), 0.52 (0.33–0.81), and 0.70 (0.50–0.99), respectively, and the test for trend was significant (*p* = 0.009). Moreover, quartile 4 and quartile 3 of total zinc and selenium intakes showed significantly lower risks of VSD and atrial septal defects (ASD) compared to the lowest quartile, and the tests for trend were significant (all *p* < 0.004). Total zinc to copper ratio and total selenium to copper ratio were inversely associated with the risks of total CHDs, VSD, and ASD (all *P* for trend <0.02). However, we observed no significant associations of total copper intake and total zinc to selenium ratio with total CHDs, VSD, and ASD. Similarly, dietary intakes of zinc, selenium, zinc to copper ratio, and selenium to copper ratio during pregnancy were inversely associated with the risks of total CHDs, VSD, and ASD, while no significant associations of dietary copper intake and dietary zinc to selenium ratio with the risks of total CHDs, VSD, and ASD were found (Table S1).

Mothers whose total zinc and selenium intakes met the RNIs had significantly lower risks of total CHDs (total zinc: OR = 0.56, 95%CI = 0.37–0.84; total selenium: OR = 0.23, 95%CI = 0.11–0.49), VSD, and ASD (Table 3). However, the fully adjusted ORs were not significant for total CHDs, VSD, and ASD associated with total copper intake meeting the RNI. Similarly, mothers whose dietary zinc and selenium intakes met the RNIs had lower risks of total CHDs, while no significant associations of dietary copper intake meeting the RNI with total CHDs, VSD, and ASD were observed (Table S2).

**Figure 1:**
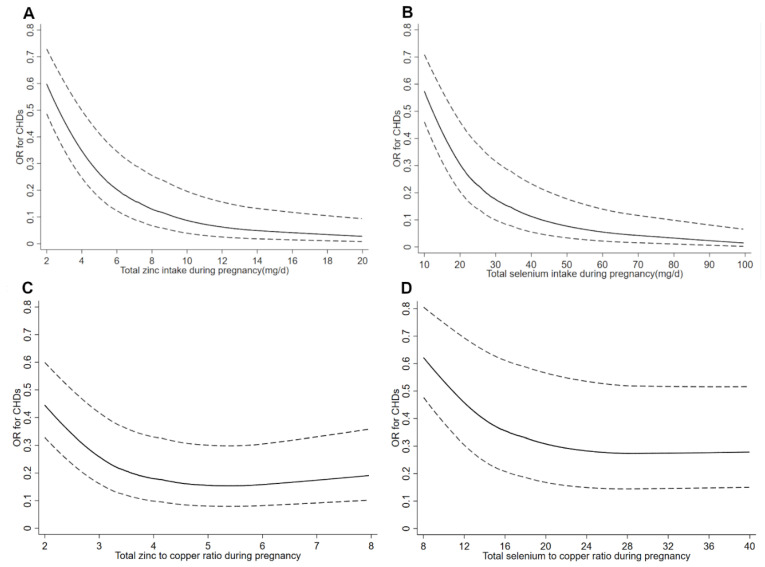
Restricted cubic spline models of total congenital heart defects (CHDs) risk associated with (**A**) total zinc intake, (**B**) total selenium intake, (**C**) total zinc to copper ratio, and (**D**) total selenium to copper ratio during pregnancy. Adjusted for total energy intake in pregnancy, socio-demographic characteristics (maternal age, residence, education, work, and parity), maternal health-related factors in the first trimester (folate/iron supplements use, passive smoking, medication use, and anemia), and dietary diversity score.

Figure 1 depicts the restricted cubic spline curves for the associations between total intakes of zinc, selenium, zinc to copper ratio, and selenium to copper ratio in pregnancy and total CHDs. The risk for total CHDs decreased with increasing intakes of total zinc and selenium in pregnancy and reached a plateau of total zinc and selenium above 15.1 mg/d and 61.7 mg/d, respectively. The risk for total CHDs decreased with increasing total zinc to copper ratio when the ratio was below 6.0 and then slightly increased when the ratio above 6.0. The risk for total CHDs decreased with increasing the total selenium to copper ratio when the ratio was below 28.4 and then slightly increased when the ratio was above 28.4. The restricted cubic spline curves for the relationships between dietary intakes of zinc, selenium, zinc to copper ratio, and selenium to copper ratio during pregnancy and total CHDs showed similar shapes as to the corresponding total intakes (Figure S1).

When introducing interaction terms into the regression models, the associations of maternal total and dietary intakes of zinc, copper, selenium, zinc to copper ratios, selenium to copper ratios, and zinc to selenium ratios with CHDs did not meaningfully vary by maternal age, residence, education, work, and folate/iron supplements use in the first trimester, and the tests for interactions were not significant (all *p* > 0.05).

**Figure 2:**
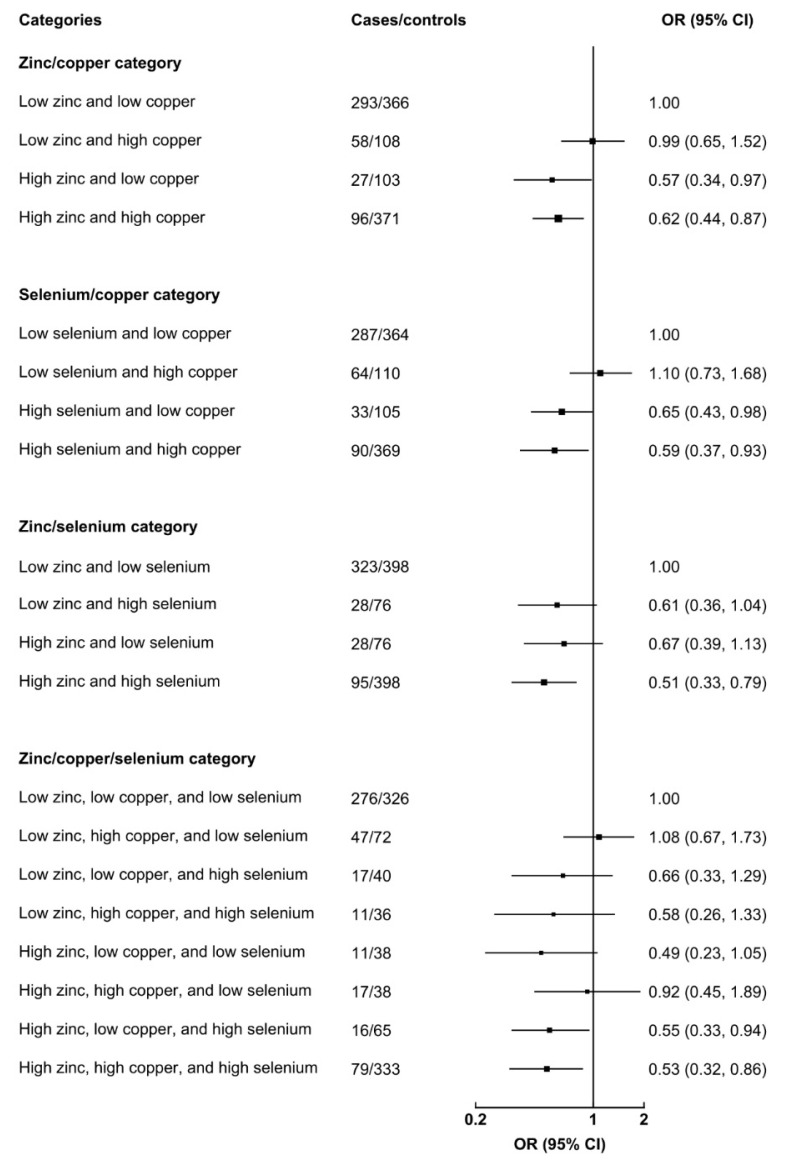
Interaction effects among total zinc, copper, and selenium intakes in pregnancy on total congenital heart defects. Adjusted for total energy intake in pregnancy, socio-demographic characteristics (maternal age, residence, education, work, and parity), maternal health-related factors in the first trimester (folate/iron supplements use, passive smoking, medication use, and anemia), and dietary diversity score. Low zinc, low copper, and low selenium indicate the intakes below medians of the control distribution, and high zinc, high copper, and high selenium indicate the intakes equal or above medians of the control distribution. The black boxes represent odds ratios, and the horizontal lines represent 95%CIs.

Although there were no significant multiplicative interactions among total zinc and copper, selenium and copper, or zinc and selenium in the regression models (all *p* > 0.05), some significant results were derived from the categorical variables as shown in Figure 2. High zinc, even with low or high copper intake, showed a lower risk for total CHDs than that of low zinc and low copper. Similarly, high selenium, even with low or high copper intake, showed a lower risk for total CHDs than that of low selenium and low copper. Compared with low zinc and low selenium, high zinc and high selenium intakes reduced the risk of total CHDs (OR = 0.51, 95%CI = 0.33–0.79). Moreover, using low zinc, low copper, and low selenium intakes as the reference, high zinc, low copper, and high selenium intakes (OR = 0.55, 95%CI = 0.33–0.97) and high zinc, high copper, and high selenium intakes (OR = 0.53, 95%CI = 0.32–0.96) showed a significantly lower risk of total CHDs. 

### 3.3. Maternal Zinc, Copper, and Selenium Supplements Uses during Pregnancy and CHDs

Maternal zinc and selenium supplements uses during pregnancy were associated with reduced risks of total CHDs (zinc supplements use: OR = 0.53, 95%CI = 0.37–0.76; selenium supplements use: OR = 0.45, 95%CI = 0.30–0.68), VSD, and ASD (Table S3). Maternal zinc supplements use during the first trimester was associated with reduced risks of total CHDs (OR = 0.58, 95%CI = 0.38–0.91) and ASD, and maternal selenium supplements use during the first trimester was associated with a lower risk of total CHDs (OR = 0.52, 95%CI = 0.31–0.85). However, we observed no significant associations of maternal copper supplements use in pregnancy and in the first trimester with CHDs.

## 4. Discussion

In the present case-control study, we observed that higher total intakes of zinc, selenium, zinc to copper ratio, and selenium to copper ratio during pregnancy were associated with reduced risks of total CHDs and the subtypes. The significantly inverse associations with CHDs were also observed for dietary intakes of zinc, selenium, zinc to copper ratio, and selenium to copper ratio during pregnancy, and maternal zinc and selenium supplements use during pregnancy and in the first trimester. Moreover, high zinc and high selenium, even with low or high copper, showed a significantly lower risk of total CHDs. To our knowledge, this is the first human study to specifically explore the relationships between maternal total, dietary, and supplemental intakes of zinc, copper, and selenium during pregnancy and CHDs.

### 4.1. Comparisons with Other Studies

To date, few human studies have explored the effects of zinc, copper, and selenium on CHDs [11,12,13,14,15,16]. The related human studies involved zinc, copper, and selenium status in blood, hair, and teeth samples among mothers, neonates, and children [11,12,13,14,15,16]. However, the results remained controversial [11,12,13,14,15,16]. One study reported an inverse association between zinc level in children’s blood and VSD [16], while another study found no associations of zinc level in maternal hair with CHDs and the subtypes [13]. There was even one study reporting higher zinc levels in maternal and neonatal blood in the CHDs cases than in the healthy controls [11]. Two studies showed positive associations of copper levels in maternal hair and children’s teeth with CHDs [13,15], while the other two studies reported no associations of copper levels in maternal and children’s blood with CHDs and VSD [14,16]. One study observed an inverse association between selenium level in maternal hair and CHDs [12], while another study showed a positive association in maternal blood [14]. The present study focused on maternal intakes of zinc, copper, and selenium during pregnancy and found significant inverse associations of zinc and selenium intakes with CHDs but no significant association for copper intake. These inconsistent results may be partially due to the differences in exposure measurement methods, sample size, study population, and genetic backgrounds. Moreover, the selenium status of an individual was largely determined by not only food sources but also the soil selenium concentration [37]. The selenium concentration in soil is generally influenced by geographical location, seasonal changes, protein content, and food processing [38]. People from different regions may have different baseline selenium statuses due to the soil selenium status, further leading to different health outcomes. Previous studies have reported that some dietary factors, such as cereal-based diets and fiber-rich foods, may inhibit zinc absorption [39] and further influence the association between zinc intake and health outcomes. Future studies integrating maternal minerals intakes and biological markers with genetic and soil factors are warranted to explore these relationships.

To our knowledge, there is only one published study involving the interactions of zinc and copper on CHDs [13]. This previous study did not observe a significant interaction between copper and zinc levels on CHDs [13], which was consistent with the finding in the present study. To our knowledge, there have been no previous studies of selenium–copper and zinc–selenium interactions on CHDs. Although no multiplicative interactions among zinc, copper, and selenium intakes during pregnancy on CHDs were observed in our study, the results of categorical variables suggested that high zinc and high selenium intakes, even with low or high copper, reduced the risk of CHDs. It seems that the simultaneous high intakes of zinc and selenium during pregnancy might have an additive effect on the association with CHDs. Given the rarity of research on the joint effects of zinc, copper, and selenium for fetal cardiovascular development, further studies are warranted to confirm and interpret these findings.

### 4.2. Possible Mechanisms

Zinc is involved in the synthesis of many lipids, nucleic acids, and proteins. Zinc deficiency could induce alterations in the distribution of connexin-43 and HNK-1 in fetal hearts and result in the occurrence of heart anomalies [5]. Zinc deficiency could also activate apoptotic and inflammatory processes and decrease TGF-β1 expression and nitric oxide synthase activity in cardiac tissue [40]. Zinc supplementation was reported to significantly downregulate protein and mRNA expression of metallothionein in the developing heart of embryos and decreased apoptosis and reduced levels of reactive oxygen species, regarded as a potential therapy for diabetic cardiac embryopathy [41]. 

Copper ions serve as an important catalytic cofactor in the redox chemistry of proteins exerting fundamental biological functions, such as cytochrome C oxidase, Cu/Zn superoxide dismutase, and ceruloplasmin. With a relatively high DNA binding affinity, copper may displace zinc ions in zinc-finger transcription factors and interfere with their functions in fetuses [42]. Inhibition of zinc-finger transcription factors, such as GATA4 and Zac1, could lead to embryonic lethality, thin ventricular walls, or abnormal looping morphogenesis of the primary heart tube [43,44]. However, no significant results were found on the associations of dietary and supplemental copper intakes with CHDs in the present study. The reason may come from the fact that few pregnant women have copper deficiency and excess in our study population. In fact, the percentages of participants with total copper intake below the RNI (0.9 mg/d) were 15.2% in the cases and 8.4% in the controls, and no participants had a total copper intake above the tolerable upper intake level (8 mg/d) in the two groups.

Selenium is essential for antioxidant enzyme activities and normal fetal development [45]. Selenium deficiency in pregnancy might contribute to congenital anomalies, including neural tube defects and orofacial clefts [46,47,48]. Selenium exposure was reported to be associated with the changes in epigenetic patterning in both human and animal studies [49,50], which may exert effects on fetal cardiovascular development. It is noteworthy to mention that the study area in Northwest China is a relatively selenium deficient area, in which pregnant women tend to have low selenium status at baseline and can benefit more from the increased intake of selenium from diet and supplements. 

Antagonisms between zinc, copper, and selenium have been shown in previous studies [18,19,20]. High zinc levels reduced the transport of copper into the blood, whereas high copper reduced zinc transport into the blood [19]. Copper was reported to negatively affect selenoprotein expression and activity via limiting UGA recoding [20]. Selenium had an antagonistic effect on zinc absorption by zinc-depleted rats, and zinc had an antagonistic effect on selenium absorption by zinc-adequate rats [18]. Given the rarity of research on the interactions of zinc, copper, and selenium on CHDs, the potential mechanisms involved need to be further explored.

### 4.3. Strengths and Limitations

The present study provides valuable evidence on the relationships between maternal total, dietary, and supplemental intakes of zinc, copper, and selenium during pregnancy and CHDs among humans. However, some limitations should be acknowledged. First, selection bias cannot be excluded because of the fact that pregnant women with CHDs fetuses tend to choose comprehensive hospitals for delivery, including the six selected hospitals in our study. Selection bias may also come from the fact that CHDs fetuses that did not survive were not included in the current study. If low maternal intakes of zinc and selenium in pregnancy increased CHDs risk and caused spontaneous and elective abortions, the relationships would be underestimated. Second, recall bias cannot be excluded because maternal information in pregnancy was recalled by participants waiting for delivery in the obstetrics departments. However, previous studies have suggested that nutrient intakes and events during pregnancy could be recalled well even after years [51,52]. To minimize bias, we made efforts to help participants recall accurately in the survey. For one thing, standard questionnaires and supporting materials such as food portion images and calendars were applied to collect information. For another, the survey was tested in a pilot study, and interviewers were rigorously trained according to the standard guides before the formal survey. Third, exposure misclassification may cause because we collected dietary information during the whole pregnancy rather than in the 3rd–8th week of gestation, the critical period of cardiac development. However, previous studies have reported that maternal dietary patterns and nutrient intakes were stable across pregnancy [24,25]. Fourth, we cannot separately assess the associations between maternal zinc, copper, and selenium intakes and other CHDs subtypes because of the limited sample size. Finally, we cannot fully exclude all other unobserved and unknown confounders and cannot reveal a real causal association.

## 5. Conclusions

The current study suggests that higher intakes of zinc and selenium from diet and supplements during pregnancy may reduce CHDs risk. This study also suggests that high intakes of zinc and selenium during pregnancy seem to have an additive effect on the association with CHDs. These findings imply the importance of promoting zinc and selenium intakes in pregnancy to reduce the incidence of CHDs in Northwest China. Future human studies with data on maternal minerals intakes, biological markers, and genetic and soil factors are warranted to confirm these findings and to elucidate underlying mechanisms.

## Figures and Tables

**Table 1:** Basic characteristics of the study participants.

	Cases (*N* = 474)	Controls (*N* = 948)	*p*
Socio-demographic characteristics, %			
Maternal age ≥ 30 years	33.5	34.2	0.812
Urban residence	66.0	71.6	0.030
Maternal education, senior high school or above	58.9	80.7	<0.001
Maternal work, farmers	49.5	21.0	<0.001
Nulliparity	57.8	80.3	<0.001
Maternal health-related factors in the first trimester, %			
Folate/iron supplements use	76.6	89.2	<0.001
Passive smoking	33.5	9.3	<0.001
Medication use	41.6	30.4	<0.001
Anemia	16.9	10.9	<0.001
Neonatal gender, male, %	52.3	49.7	0.348
Dietary diversity score, median (25th percentile, 75th percentile)	5.0 (3.0, 6.0)	6.0 (4.0, 8.0)	<0.001
Daily components intake during pregnancy, median (25th percentile, 75th percentile)			
Total energy, kcal	1753.2 (1452.4, 2086.1)	1907.1 (1563.3, 2415.9)	0.001
Total zinc, mg	5.1 (3.2, 7.3)	7.2 (5.1, 10.9)	<0.001
Dietary zinc, mg	4.7 (3.1, 6.8)	6.4 (4.6, 9.1)	<0.001
Total copper, mg	1.6 (1.1, 2.1)	2.0 (1.4, 2.7)	<0.001
Dietary copper, mg	1.6 (1.1, 2.1)	1.9 (1.2, 2.5)	<0.001
Total selenium, mg	23.2 (15.4, 32.8)	32.5 (22.7, 46.6)	<0.001
Dietary selenium, mg	22.7 (15.1, 32.6)	30.9 (21.9, 43.7)	<0.001
Total zinc to copper ratio	3.2 (2.4, 4.4)	3.8 (3.1, 4.8)	<0.001
Dietary zinc to copper ratio	3.1 (2.4, 4.2)	3.6 (3.0, 4.5)	<0.001
Total selenium to copper ratio	14.7 (10.8, 20.7)	16.7 (13.5, 21.6)	<0.001
Dietary selenium to copper ratio	14.7 (10.8, 20.9)	17.2 (13.7, 22.7)	<0.001
Total zinc to selenium ratio	0.21 (0.18, 0.25)	0.22 (0.19, 0.27)	0.280
Dietary zinc to selenium ratio	0.21 (0.18, 0.25)	0.21 (0.18, 0.23)	0.270

Categorical variables are compared between groups by χ^2^ test, and continuous variables are compared between groups by Mann–Whitney *U* test.

**Table 2:** Quartiles of maternal total zinc, copper, and selenium intakes during pregnancy and congenital heart defects.

	Cutoffs	Total CHDs (*N*_cases_ = 474)			VSD (*N*_cases_ = 223)	ASD (*N*_cases_ = 218)
		Cases/Controls	Unadjusted OR (95%CI)	Adjusted OR (95%CI) ^1^	Adjusted OR (95%CI) ^1^	Adjusted OR (95%CI) ^1^
Total zinc intake (mg/d)						
Quartile 1	<5.09	241/236	1	1	1	1
Quartile 2	5.09–7.21	110/238	0.45 (0.34, 0.60)	0.65 (0.45, 0.94)	0.71 (0.39, 1.27)	0.61 (0.36, 1.03)
Quartile 3	7.21–10.86	82/237	0.34 (0.25, 0.46)	0.57 (0.36, 0.91)	0.53 (0.32, 0.86)	0.55 (0.32, 0.96)
Quartile 4	≥10.86	41/237	0.17 (0.12, 0.25)	0.22 (0.12, 0.42)	0.14 (0.05, 0.35)	0.21 (0.09, 0.48)
*p* for trend ^2^			<0.001	<0.001	0.002	0.001
Total copper intake (mg/d)						
Quartile 1	<1.37	153/237	1	1	1	1
Quartile 2	1.37–1.95	167/237	1.10 (0.82, 1.46)	1.26 (0.90, 1.77)	1.30 (0.84, 2.03)	1.19 (0.77, 1.85)
Quartile 3	1.95–2.70	103/237	0.66 (0.48, 0.90)	1.24 (0.82, 1.85)	1.37 (0.81, 2.31)	1.13 (0.68, 1.88)
Quartile 4	≥2.70	51/237	0.33 (0.23, 0.47)	0.66 (0.38, 1.16)	0.77 (0.37, 1.64)	0.54 (0.26, 1.14)
*p* for trend ^2^			<0.001	0.534	0.943	0.344
Total selenium intake (mg/d)						
Quartile 1	<22.68	227/237	1	1	1	1
Quartile 2	22.68–32.45	124/237	0.55 (0.41, 0.73)	0.70 (0.50, 0.99)	0.64 (0.40, 1.01)	0.71 (0.45, 1.13)
Quartile 3	32.45–46.61	76/237	0.33 (0.24, 0.46)	0.52 (0.33, 0.81)	0.47 (0.26, 0.85)	0.55 (0.31, 0.98)
Quartile 4	≥46.51	47/237	0.21 (0.14, 0.30)	0.29 (0.15, 0.54)	0.13 (0.05, 0.34)	0.25 (0.11, 0.59)
*P* for trend ^2^			<0.001	0.009	<0.001	0.003
Total zinc to copper ratio						
Quartile 1	<3.10	225/237	1	1	1	1
Quartile 2	3.10–3.84	92/237	0.41 (0.30, 0.55)	0.59 (0.41, 0.84)	0.64 (0.41, 1.00)	0.67 (0.43, 1.05)
Quartile 3	3.84–4.81	69/237	0.31 (0.22, 0.42)	0.43 (0.29, 0.63)	0.42 (0.26, 0.70)	0.40 (0.24, 0.67)
Quartile 4	≥4.81	88/237	0.39 (0.29, 0.53)	0.58 (0.41, 0.82)	0.49 (0.30, 0.78)	0.66 (0.44, 0.98)
*p* for trend ^2^			<0.001	<0.001	<0.001	0.017
Total selenium to copper ratio						
Quartile 1	<13.48	197/237	1	1	1	1
Quartile 2	13.48–16.68	101/237	0.51 (0.38, 0.69)	0.65 (0.46, 0.92)	0.58 (0.37, 0.91)	0.96 (0.62, 1.48)
Quartile 3	16.68–21.61	75/237	0.38 (0.28, 0.52)	0.45 (0.31, 0.65)	0.36 (0.22, 0.59)	0.48 (0.29, 0.78)
Quartile 4	≥21.61	101/237	0.51 (0.38, 0.69)	0.65 (0.46, 0.93)	0.48 (0.30, 0.77)	0.70 (0.44, 1.10)
*p* for trend ^2^			<0.001	0.002	<0.001	0.019
Total zinc to selenium ratio						
Quartile 1	<0.19	139/237	1	1	1	1
Quartile 2	0.19–0.22	127/237	0.91 (0.68, 1.23)	0.86 (0.60, 1.22)	0.94 (0.59, 1.50)	0.88 (0.56, 1.38)
Quartile 3	0.22–0.27	119/237	0.86 (0.63, 1.16)	1.02 (0.71, 1.45)	1.34 (0.84, 2.14)	0.99 (0.63, 1.56)
Quartile 4	≥0.27	89/237	0.64 (0.46, 0.88)	0.84 (0.58, 1.23)	1.07 (0.65, 1.76)	0.94 (0.59, 1.50)
*p* for trend ^2^			0.008	0.588	0.451	0.925

ASD—atrial septal defects; CHDs—congenital heart defects; VSD—ventricular septal defects. ^1^ Adjusted for total energy intake in pregnancy, socio-demographic characteristics (maternal age, residence, education, work, and parity), maternal health-related factors in the first trimester (folate/iron supplements use, passive smoking, medication use, and anemia), and dietary diversity score. ^2^ *p* for trend across quartiles is calculated using the median for each quartile as a continuous variable.

**Table 3:** Maternal total zinc, copper, and selenium intakes categorized by the recommended nutrient intakes (RNIs) during pregnancy and congenital heart defects.

	Total CHDs (*N*_cases_ = 474)			VSD (*N*_cases_ = 223)	ASD (*N*_cases_ = 218)
	Cases/Controls	Unadjusted OR (95%CI)	Adjusted OR (95%CI) ^1^	Adjusted OR (95%CI) ^1^	Adjusted OR (95%CI) ^1^
Total zinc intake					
Below the RNI	406/639	1	1	1	1
Met the RNI	68/309	0.35 (0.26, 0.46)	0.56 (0.37, 0.84)	0.46 (0.26, 0.82)	0.53 (0.31, 0.91)
*p*		<0.001	0.006	0.009	0.021
Total copper intake					
Below the RNI	72/80	1	1	1	1
Met the RNI	402/868	0.51 (0.37, 0.72)	0.96 (0.63, 1.47)	0.91 (0.53, 1.55)	0.85 (0.50, 1.44)
*p*		<0.001	0.860	0.728	0.540
Total selenium intake					
Below the RNI	460/837	1	1	1	1
Met the RNI	14/111	0.23 (0.13, 0.40)	0.23 (0.11, 0.49)	0.17 (0.05, 0.51)	0.18 (0.07, 0.47)
*p*		<0.001	<0.001	0.002	<0.001

ASD—atrial septal defects; CHDs—congenital heart defects; VSD—ventricular septal defects; RNI—recommended nutrients intake. ^1^ Adjusted for total energy intake in pregnancy, socio-demographic characteristics (maternal age, residence, education, work, and parity), maternal health-related factors in the first trimester (folate/iron supplements use, passive smoking, medication use, and anemia), and dietary diversity score.

## Data Availability

The data present in this study are available on request from the corresponding authors.

## Supplementary Materials

The following supporting information can be downloaded at https://www.mdpi.com/article/10.3390/nu14051055/s1, Figure S1: Restricted cubic spline models of total congenital heart defects (CHDs) risk associated with (A) dietary zinc intake, (B) dietary selenium intake, (C) dietary zinc to copper ratio, and (D) dietary selenium to copper ratio during pregnancy. Models are adjusted for total energy intake in pregnancy, socio-demographic characteristics (maternal age, residence, education, work, and parity), maternal health-related factors in the first trimester (folate/iron supplements use, passive smoking, medication use, and anemia), and dietary diversity score. Models are additionally adjusted for maternal supplements uses of zinc, copper, and selenium in the associations between dietary intakes of corresponding minerals and CHDs; Table S1: Quartiles of maternal dietary zinc, copper, and selenium intakes during pregnancy and congenital heart defects; Table S2: Maternal dietary zinc, copper, and selenium intakes categorized by the recommended nutrient intakes (RNIs) during pregnancy and congenital heart defects; Table S3: Maternal zinc, copper, and selenium supplements use during pregnancy and congenital heart defects.
