# Supplementary material for: Maternal Zinc, Copper, and Selenium Intakes during Pregnancy and Congenital Heart Defects
Source: Nutrients. 2022 Mar 2;14(5):1055. doi: 10.3390/nu14051055 (PMC8912688; doi:10.3390/nu14051055)
Supplement: Supplementary file 1 [file nutrients-14-01055-s001.zip › nutrients-1547451-supplementary.pdf]

Table S1. Quartiles of maternal dietary zinc, copper, and selenium intakes during pregnancy and congenital heart defects.

	Cutoffs	Total CHDs (N_{cases}=474)			VSD (N_{cases}=223)	ASD (N_{cases}=218)
		Cases/Controls	Adjusted OR (95%CI) [1]	Adjusted OR (95%CI) [1]	Adjusted OR (95%CI) [1]	Adjusted OR (95%CI) [1]
Dietary zinc intake (mg/d)						
Quartile 1	<4.64	231/237	1	1	1	1
Quartile 2	4.64-6.38	101/237	0.44 (0.33, 0.59)	0.56 (0.37, 0.83)	0.56 (0.29, 1.10)	0.54 (0.32, 0.89)
Quartile 3	6.38-9.08	83/237	0.36 (0.26, 0.49)	0.56 (0.33, 0.93)	0.52 (0.31, 0.88)	0.51 (0.27, 0.95)
Quartile 4	≥9.08	59/237	0.26 (0.18, 0.36)	0.32 (0.15, 0.69)	0.23 (0.08, 0.66)	0.33 (0.12, 0.91)
P for trend [2]			<0.001	0.007	0.020	0.029
Dietary copper intake (mg/d)						
Quartile 1	<1.25	142/237	1	1	1	1
Quartile 2	1.25-1.86	158/237	1.11 (0.83, 1.49)	1.37 (0.96, 1.94)	1.39 (0.88, 2.21)	1.18 (0.75, 1.86)
Quartile 3	1.86-2.49	114/237	0.80 (0.59, 1.09)	1.64 (1.08, 2.49)	2.09 (1.21, 3.60)	1.54 (0.91, 2.61)
Quartile 4	≥2.49	60/237	0.42 (0.30, 0.60)	0.94 (0.54, 1.65)	1.23 (0.58, 2.64)	0.86 (0.42, 1.78)
P for trend [2]			<0.001	0.470	0.125	0.709
Dietary selenium intake (mg/d)						
Quartile 1	<21.90	224/237	1	1	1	1
Quartile 2	21.90-30.89	113/237	0.50 (0.38, 0.67)	0.66 (0.46, 0.95)	0.58 (0.36, 0.93)	0.69 (0.43, 1.10)
Quartile 3	30.89-43.70	84/237	0.38 (0.28, 0.51)	0.57 (0.36, 0.90)	0.49 (0.27, 0.89)	0.56 (0.31, 1.01)
Quartile 4	≥43.70	53/237	0.24 (0.17, 0.34)	0.33 (0.17, 0.63)	0.15 (0.06, 0.40)	0.26 (0.11, 0.64)
P for trend [2]			<0.001	0.001	<0.001	0.006
Dietary zinc to copper ratio						
Quartile 1	<2.99	216/237	1	1	1	1
Quartile 2	2.99-3.60	86/237	0.40 (0.29, 0.54)	0.60 (0.42, 0.85)	0.70 (0.45, 1.10)	0.72 (0.46, 1.13)
Quartile 3	3.60-4.47	75/237	0.35 (0.25, 0.48)	0.55 (0.37, 0.79)	0.53 (0.32, 0.87)	0.57 (0.35, 0.93)
Quartile 4	≥4.47	97/237	0.45 (0.33, 0.61)	0.61 (0.43, 0.87)	0.50 (0.31, 0.79)	0.71 (0.45, 1.11)
P for trend [2]			<0.001	0.003	0.001	0.079
Dietary selenium to copper ratio						
Quartile 1	<13.68	204/237	1	1	1	1
Quartile 2	13.68-17.18	103/237	0.50 (0.37, 0.68)	0.65 (0.46, 0.92)	0.63 (0.41, 0.99)	0.93 (0.61, 1.42)
Quartile 3	17.18-21.71	80/237	0.39 (0.29, 0.54)	0.50 (0.35, 0.73)	0.41 (0.25, 0.66)	0.50 (0.31, 0.81)
Quartile 4	≥21.71	87/237	0.43 (0.31, 0.58)	0.54 (0.38, 0.78)	0.41 (0.25, 0.66)	0.55 (0.34, 0.88)
P for trend [2]			<0.001	<0.001	<0.001	0.002
Dietary zinc to selenium ratio						
Quartile 1	<0.18	121/237	1	1	1	1
Quartile 2	0.18-0.21	115/237	0.83 (0.60, 1.14)	0.82 (0.56, 1.19)	0.68 (0.41, 1.14)	0.98 (0.61, 1.58)
Quartile 3	0.21-0.23	118/237	0.85 (0.62, 1.17)	0.86 (0.59, 1.26)	1.04 (0.64, 1.69)	0.88 (0.54, 1.44)
Quartile 4	≥0.23	120/237	1.24 (0.92, 1.67)	0.99 (0.63, 1.56)	1.02 (0.65, 1.60)	0.98 (0.65, 1.60)
P for trend [2]			0.139	0.230	0.310	0.190

ASD, atrial septal defects; CHDs, congenital heart defects; VSD, ventricular septal defects.

[1] Models are adjusted for total energy intake during pregnancy, socio-demographic characteristics (maternal age, residence, education, work, and parity), maternal health-related factors in the first trimester (folate/iron supplements use, passive smoking, medication use, and anemia), and dietary diversity score. Models are additionally adjusted for maternal supplements uses of zinc, copper, and selenium in the associations between dietary intakes of corresponding minerals and CHDs.

[2] *P* for trend across quartiles is calculated using the median for each quartile as a continuous variable.

Table S2. Maternal dietary zinc, copper, and selenium intakes categorized by the recommended nutrient intakes (RNIs) during pregnancy and congenital heart defects.

	Total CHDs (N_{cases}=474)			VSD (N_{cases}=223)	ASD (N_{cases}=218)
	**Cases/Controls**	**Adjusted OR (95%CI) [1]**	**Adjusted OR (95%CI) [1]**	**Adjusted OR (95%CI) [1]**	**Adjusted OR (95%CI) [1]**
Dietary zinc intake					
Below the RNI	423/736	1	1	1	1
Met the RNI	51/212	0.42 (0.30, 0.58)	0.67 (0.49, 0.93)	0.54 (0.26, 1.12)	0.82 (0.42, 1.60)
P		<0.001	0.016	0.099	0.565
Dietary copper intake					
Below the RNI	74/105	1	1	1	1
Met the RNI	400/843	0.69 (0.41, 1.17)	1.24 (0.83, 1.87)	1.22 (0.72, 2.06)	1.05 (0.63, 1.74)
P		0.166	0.295	0.451	0.860
Dietary selenium intake					
Below the RNI	463/852	1	1	1	1
Met the RNI	11/96	0.21 (0.11, 0.40)	0.18 (0.08, 0.42)	0.15 (0.05, 0.51)	0.18 (0.06, 0.50)
P		<0.001	<0.001	0.002	0.001

ASD, atrial septal defects; CHDs, congenital heart defects; VSD, ventricular septal defects.

[1] Models are adjusted for total energy intake during pregnancy, socio-demographic characteristics (maternal age, residence, education, work, and parity), maternal health-related factors in the first trimester (folate/iron supplements use, passive smoking, medication use, and anemia), and dietary diversity score. Models are additionally adjusted for maternal supplements uses of zinc, copper, and selenium in the associations between dietary intakes of corresponding minerals and CHDs.

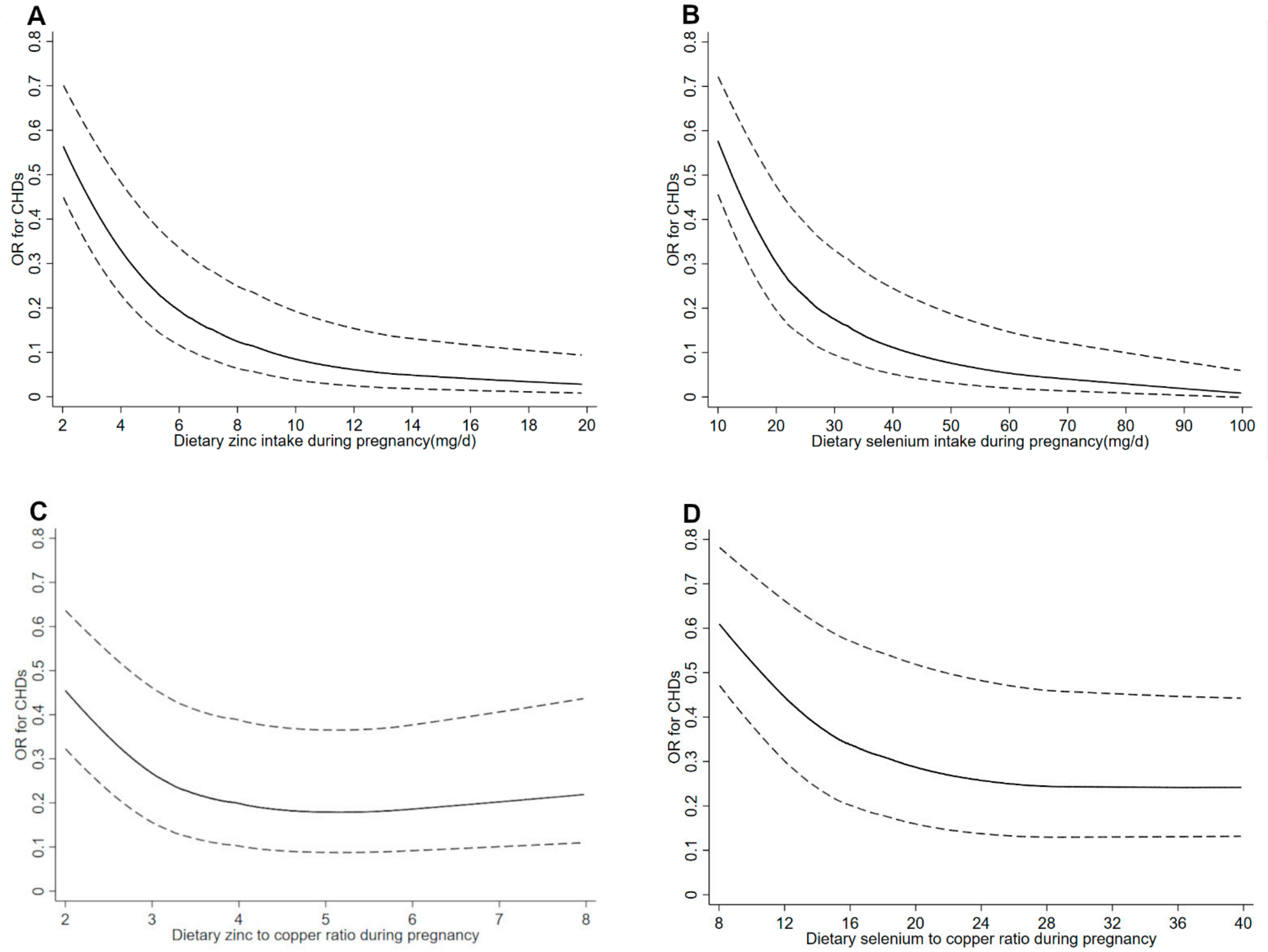

Figure S1. Restricted cubic spline models of total congenital heart defects (CHDs) risk associated with (A) dietary zinc intake, (B) dietary selenium intake, (C) dietary zinc to copper ratio, and (D) dietary selenium to copper ratio during pregnancy. Models are adjusted for total energy intake during pregnancy, socio-demographic characteristics (maternal age, residence, education, work, and parity), maternal health-related factors in the first trimester (folate/iron supplements use, passive smoking, medication use, and anemia), and dietary diversity score. Models are additionally adjusted for maternal supplements uses of zinc, copper, and selenium in the associations between dietary intakes of corresponding minerals and CHDs.

Table S3. Maternal zinc, copper, and selenium supplements uses during pregnancy and congenital heart defects.

	**Total CHDs (N_{cases}=474)**		**VSD (N_{cases}=223)**		**ASD (N_{cases}=218)**	
	**OR (95%CI)**	***P***	**OR (95%CI)**	***P***	**OR (95%CI)**	***P***
Zinc supplements use during pregnancy	0.53 (0.37, 0.76)	0.001	0.51 (0.31, 0.82)	0.006	0.43 (0.26, 0.69)	0.001
Zinc supplements use in the first trimester	0.58 (0.38, 0.91)	0.016	0.62 (0.34, 1.13)	0.118	0.49 (0.27, 0.91)	0.023
Copper supplements use during pregnancy	0.52 (0.26, 1.04)	0.065	0.62 (0.24, 1.63)	0.332	0.56 (0.22, 1.48)	0.244
Copper supplements use in the first trimester	0.46 (0.15, 1.43)	0.180	0.49 (0.11, 2.22)	0.357	0.52 (0.11, 2.51)	0.416
Selenium supplements use during pregnancy	0.45 (0.30, 0.68)	<0.001	0.46 (0.26, 0.80)	0.006	0.38 (0.22, 0.66)	0.001
Selenium supplements use in the first trimester	0.52 (0.31, 0.85)	0.010	0.59 (0.31, 1.13)	0.113	0.54 (0.27, 1.05)	0.071

Models are adjusted for total energy intake during pregnancy, socio-demographic characteristics (maternal age, residence, education, work, and parity), maternal health-related factors in the first trimester (folate/iron supplements use, passive smoking, medication use, and anemia), and dietary diversity score. Models are additionally adjusted for maternal dietary intakes of zinc, copper, and selenium in the associations between supplements uses of corresponding minerals and CHDs.
